# Midgut transcriptomic responses to dengue and chikungunya viruses in the vectors *Aedes albopictus* and *Aedes malayensis*

**DOI:** 10.1038/s41598-023-38354-9

**Published:** 2023-07-12

**Authors:** Cassandra M. Modahl, Avisha Chowdhury, Dolyce H. W. Low, Menchie C. Manuel, Dorothée Missé, R. Manjunatha Kini, Ian H. Mendenhall, Julien Pompon

**Affiliations:** 1 Department of Biological Sciences, National University of Singapore, Singapore, Singapore; 2 Programme in Emerging Infectious Diseases, Duke-NUS Medical School, Singapore, Singapore; 3 Present Address: Department of Pharmacology, Yong Loo Lin School of Medicine, National University of Singapore, Singapore, Singapore; 4 Present Address: Liverpool School of Tropical Medicine, Liverpool, U.K.; 5 Present Address: Toronto Centre for Liver Disease, Toronto General Hospital, University Health Network, University of Toronto, Toronto, Canada; 6 Present Address: MIVEGEC, Univ. Montpellier, IRD, CNRS, Montpellier, France

**Keywords:** Viral infection, Dengue virus, Alphaviruses

## Abstract

Dengue (DENV) and chikungunya (CHIKV) viruses are among the most preponderant arboviruses. Although primarily transmitted through the bite of *Aedes aegypti* mosquitoes, *Aedes albopictus* and *Aedes malayensis* are competent vectors and have an impact on arbovirus epidemiology. Here, to fill the gap in our understanding of the molecular interactions between secondary vectors and arboviruses, we used transcriptomics to profile the whole-genome responses of *A. albopictus* to CHIKV and of *A. malayensis* to CHIKV and DENV at 1 and 4 days post-infection (dpi) in midguts. In *A. albopictus*, 1793 and 339 genes were significantly regulated by CHIKV at 1 and 4 dpi, respectively. In *A. malayensis*, 943 and 222 genes upon CHIKV infection, and 74 and 69 genes upon DENV infection were significantly regulated at 1 and 4 dpi, respectively. We reported 81 genes that were consistently differentially regulated in all the CHIKV-infected conditions, identifying a CHIKV-induced signature. We identified expressed immune genes in both mosquito species, using a de novo assembled midgut transcriptome for *A. malayensis*, and described the immune architectures. We found the JNK pathway activated in all conditions, generalizing its antiviral function to Aedines. Our comprehensive study provides insight into arbovirus transmission by multiple *Aedes* vectors.

## Introduction

Arboviruses (arthropod-borne viruses) are a global public health burden^1,2^. Dengue viruses (DENV) from the *Flaviviridae* family and chikungunya virus (CHIKV) from the *Togaviridae* family are among the most prevalent arboviruses. Both viruses are now endemic to subtropical and tropical areas of Asia, the Americas, Oceania and Africa, and cause sporadic epidemics in Europe and North America^3,4^. Climate change modeling forecasts an expansion of arbovirus geographic distribution into temperate regions, which have previously been relatively spared^5,6^. Currently, DENV infects an estimated 400 million people yearly, causing symptoms ranging from flu-like to severe hemorrhage, leading to shock and death for approximately 25,000 people^7^. CHIKV emerged as a global pathogen in 2004 and has ever since infected more than 6 million people, causing flu-like symptoms associated with severe polyarthralgia, in some cases lasting for years and drastically impairing life quality^8^. Unfortunately, there is no effective curative treatment for these arbovirus infections, only a sub-effective vaccine with associated safety risks for DENV^9,10^, and no licensed vaccine against CHIKV. Vector control remains the primary intervention to limit arbovirus spread and is widely deployed. However, current vector control strategies have modest efficacy in preventing epidemics and limited success against container-ovipositing species^11–13^. A better understanding of the molecular mechanisms that enable viral transmission by mosquito vectors will help to design novel interventions.

Both DENV and CHIKV are transmitted by *Aedes* mosquitoes, mainly by the species *Aedes aegypti*^7,8^. However, multiple *Aedes* species have long been known to be susceptible to DENV and CHIKV infections^14^ and can act as vectors of importance, as exemplified by the mild dengue and chikungunya epidemics that occurred in areas without *A. aegypti*^8,15–19^. For instance, *Aedes albopictus* is sufficient to maintain endemic dengue in La Réunion island^20,21^, is responsible for sporadic outbreaks of increasing intensity in Southern Europe^22–24^, and caused an outbreak in an urban park in Japan^18^. For CHIKV, its envelope protein evolved to increase transmission by *A. albopictus* without compromising *A. aegypti* vector competence^25^, causing an unprecedented epidemic spanning the Indian Ocean region, East Africa and India^3^. Beside *A. albopictus,* our group identified *Aedes malayensis*, a species closely related to *A. albopictus* and distributed throughout Southeast Asia^26^, as a competent vector for both DENV and CHIKV^27^. Vector capacity of *A. albopictus* and *A. malayensis* is further supported by their anthropophilic feeding habits^28,29^. While evidence for the vector status of these two peridomestic *Aedes* species is mounting, there is a dearth of knowledge on the molecular mechanisms that regulate DENV and CHIKV infection in these secondary vectors.

Arbovirus transmission occurs when viruses imbibed during a mosquito bite on an infectious host propagate from the midgut to the salivary glands, from where they are salivated into the skin of other hosts during subsequent bites^30^. The initial midgut infection (the first barrier) that determines transmission success is strongly controlled by immunity^30,31^. Mosquitoes mount a potent immune response to infection through the canonical immune pathways, namely, Toll (TOLLPATH), Immune-Deficiency (IMDPATH) and Janus Kinase/Signal Transduction and Activators of Transcription (JAKSTAT)^32,33^. Recently, we added the c-Jun *N*-terminal Kinase (JNK) pathway as another immune arm with broad antiviral activity against DENV, Zika virus and CHIKV^34^. The multiple immune pathways share a common architecture for activation^35^. Pathogens are detected by pathogen recognition receptors (PRR), mainly gram-negative binding (GNBP) and peptidoglycan receptor-like proteins (PGRP). The activation signal is transferred through Spaetzle proteins (SPZ) or directly to transmembrane receptors such as Toll and IMD receptors, which trigger a cytoplasmic biochemical cascade resulting in the activation and nuclear translocation of transcription factors like Rel (REL) for TOLLPATH and IMDPATH, STAT for JAKSTAT or Kayak for JNK. The signaling by PRR and through the biochemical cascade are regulated by proteases such as CLIP-domain serine proteases (CLIP) and serine protease inhibitors (SRPN). Translocated transcription factors then upregulate antiviral effectors including anti-microbial peptides (AMP), Thioester-containing proteins (TEP), C-type lectins (CTL), fibrinogen-related proteins (FREP), galectins (GALE), lysozymes (LYS), MD2-like proteins (ML), scavenger receptors (SCR), catalases (CAT), superoxide dismutases (SOD) and peroxidases (HPX), the last three acting by regulating reactive oxygen species (ROS). Moreover, RNAi limits arbovirus infections by degrading viral RNA genomes through siRNA regulator proteins (SRRP)^36,37^. Viral infection can also be restricted by autophagy (APHAG), by apoptosis mediated by caspases (CASP), caspase activators (CASPA) and inhibitors of apoptosis (IAP), and by melanization triggered by prophenoloxidases (PPO). The resulting multimodal immunity restricts DENV and CHIKV as early as 3 days post-infection (dpi)^38–44^.

Most studies on mosquito antiviral immunity have been conducted with *A. aegypti*. Our knowledge regarding the immune response in other *Aedes* vectors is strikingly deficient. Only a few transcriptomic studies have been conducted with *A. albopictus,* and, to our knowledge, none has been performed with *A. malayensis* or any other *Aedes* vector. In *A. albopictus*, a previous study showed that DENV infection induced a low number of differentially expressed genes (DEGs) at 1 and 5 dpi in midguts, but only one DEG at 1 dpi was related to immunity^45^. Another study with DENV and *A. albopictus* identified 18 upregulated DEGs with a majority of AMPs, and 13 downregulated DEGs including immune pathway components such as *Myd88* and *STAT* at 1 dpi^46^. For CHIKV, only one study looked at transcriptomic regulation in *A. albopictus*. The results show 25 DEGs primarily related to metabolism and none to immunity in midguts at 2 dpi, although these regulations could not be confirmed by RT-qPCR^47^. Despite the growing interest in *A. albopictus* and *A. malayensis*, there is a clear lack of understanding of their basic molecular responses to DENV and CHIKV.

Here, to bridge the knowledge gap in our understanding of the molecular response and immunity in secondary vectors, we provide a comprehensive account of the transcriptomic responses at 1 and 4 dpi with CHIKV in both *A. albopictus* and *A. malayensis*, and with DENV in *A. malayensis*. We deployed high-throughput sequencing and validate gene expressions by RT-qPCR in different mosquito batches. We describe infection-induced gene regulations using the chromosome-level *A. albopictus* genome assembly and a de novo assembled *A. malayensis* midgut transcriptome, as this species’ genome is not available. By providing insight regarding the transcriptomic responses to two arboviruses in two relevant *Aedes* vectors, our study expands our understanding of arbovirus transmission and identifies common targets to block mosquito transmission of arboviruses.

## Results

### Transcriptome regulations by CHIKV and DENV in *A. albopictus* and *A. malayensis* midguts at 1 and 4 dpi

To determine the transcriptomic response to arboviral infections in midguts of secondary vectors, *A. albopictus* mosquitoes were orally infected with 10^7^ pfu/ml of CHIKV and *A. malayensis* mosquitoes were orally infected with either the same concentration of CHIKV or 2 × 10^7^ pfu/ml of DENV. Both blood inoculum are within the high-end of viremia measured in patients^48,49^ and resulted in 100% infected mosquitoes^34^. Controls were fed non-infectious blood. Viruses and mosquitoes were sympatric as they were all collected in Singapore, and the CHIKV strain collected before 2010 did not possess the envelope mutation that enhances *A. albopictus* infection^50^. As *Wolbachia* can influence mosquito susceptibility to arboviruses^51^, we conducted a PCR detection assay and did not find the bacteria in our colonies (Fig. S1).

**Figure 1:**
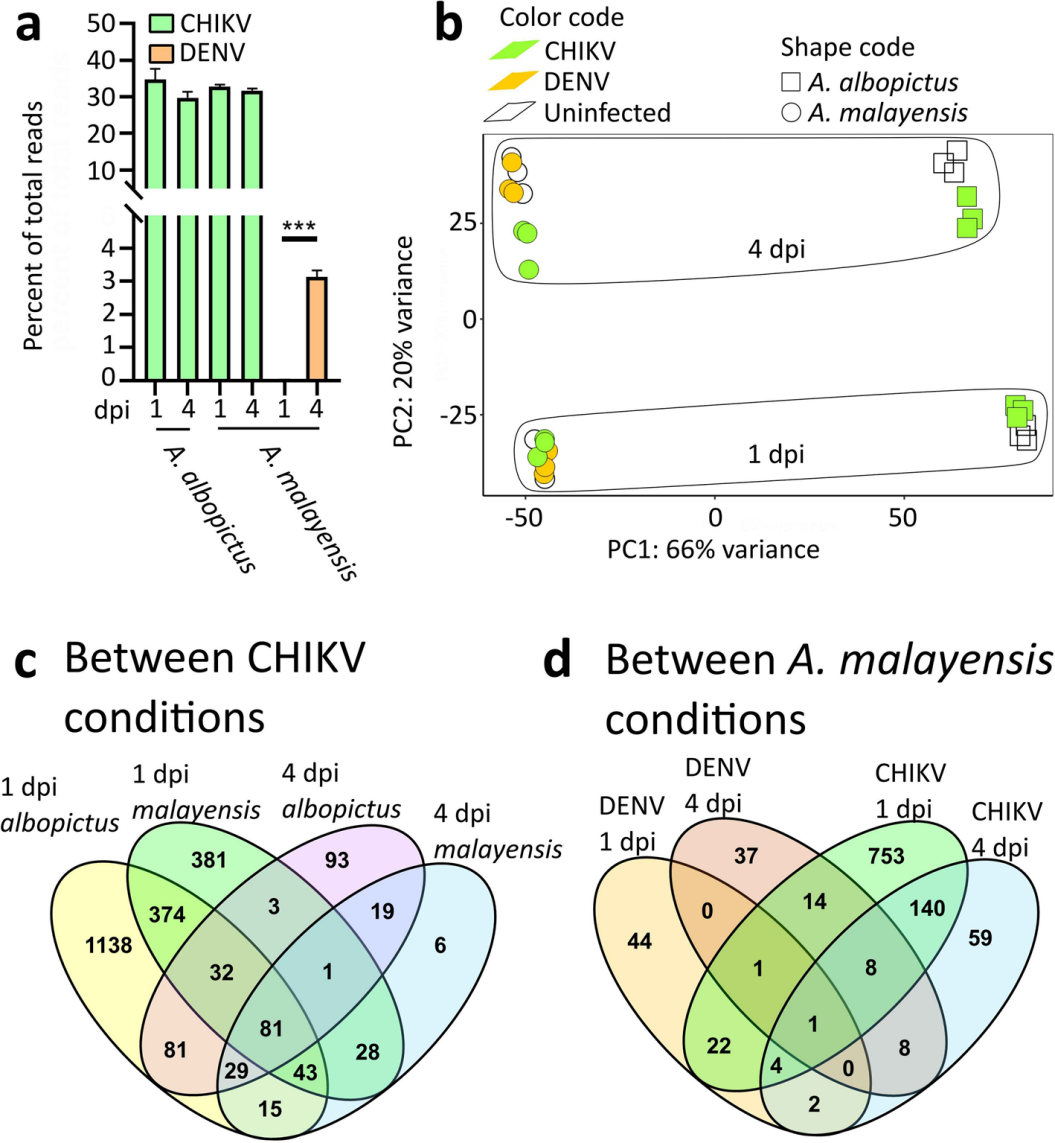
The midgut transcriptomic responses of *A. albopictus* to CHIKV and *A. malayensis* to CHIKV and DENV at 1 and 4 dpi. (**a**) Quantification of viral reads. ***, *p* < 0.001 as determined by unpaired t-test. (**b**) Principal Component Analysis (PCA) using DESeq2 normalized gene expression values. (**c**, **d**) Venn diagrams of DEGs with conditions with CHIKV infection (**c**) and with conditions with *A. malayensis.*

We then performed high-throughput RNA-sequencing on three replicates of 20 pooled midguts collected at 1 and 4 dpi for each virus and mosquito combination. We selected these two time points to cover the initial response to infection at 1 dpi and the established infection at 4 dpi. We obtained between 38 and 52 million reads per sample (Table S1). After quality filtering, we confirmed mosquito species and virus infection by mapping reads to species-specific cytochrome c oxidase (COI) genes and each viral genome, respectively (Table S1). We also quantified the percentage of viral reads and observed that CHIKV reads were more abundant than DENV and had plateaued at 1 dpi in both mosquito species, whereas DENV reads increased between 1 and 4 dpi (Fig. 1a). Since CHIKV and DENV have different patterns of infection kinetics^52,53^, this should be taken into account when interpreting gene regulations.

Using the most recent genome assembles for *A. albopictus*^54^ (AalbFP1.0) and *A. aegypti*^55^ (AaegL5.0) (*A. malayensis* genome has not been assembled), we mapped reads from *A. albopictus* and *A. malayensis* and calculated DEGs between infected samples and time-matched non-infected controls. To select the best genome approach and validate DEGs, we quantified expression levels of several genes at 4 dpi by RT-qPCR in midguts of separate mosquito batches for both species. For both *A. albopictus* and *A. malayensis*, DEGs obtained by mapping on the *A. albopictus* genome correlated best with qPCR data (Fig. S2 and S3) and were further analyzed. Of note, *A. malayensis* genes do not have IDs and hereafter, are identified from *A. albopictus* orthologs.

To identify similarities in the transcriptomes between our different conditions, we performed a principal component analysis (PCA) on normalized gene expression values (Fig. 1b). Interestingly, virus infection was not the main source of transcriptome variation, mosquito species and time post blood-feeding were responsible for the greatest variance. The PC1 explained 66% of the total variance and segregated the mosquito species, and the PC2 explained 20% of the total variance and separated the time of collection.

Upon CHIKV infection in *A. albopictus*, there were 1,793 DEGs at 1 dpi and 339 at 4 dpi (Table S2). Upon CHIKV infection in *A. malayensis*, there were 943 DEGs at 1 dpi and 222 at 4 dpi. Upon DENV infection in *A. malayensis*, there were 74 DEGs at 1 dpi and 69 at 4 dpi.

Among all the CHIKV infection conditions (i.e., both mosquito species at 1 and 4 dpi), we identified shared DEGs (Fig. 1c). First, we found 81 DEGs common to all conditions and that 79 were upregulated in all conditions (Table S2). The top eight most regulated were highly expressed (> 48 fold) and included two uncharacterized genes (*AALFPA_051205*, *AALFPA_066142*), one gene putatively involved in antiviral response (*AALFPA_05206*), one serine-rich protein kinase (*AALFPA_068585*), one deubiquitinase (*AALFPA_074126*), one cytochrome P450 (*AALFPA_062825*), one homologue of *hunchback* (*AALFPA_070528*) and one homolog of *canoe* (*AALFPA_068948*). Second, we looked at the effect of time post infection within mosquito species. We found that 223 DEGs were conserved in *A. albopictus* between 1 and 4 dpi (Table S2), representing 12% and 66% of all DEGs for each dpi, respectively. We observed that 153 DEGs were common in *A. malayensis* between 1 and 4 dpi, representing 16% and 69% of within-dpi DEGs, respectively. Third, we reported the shared DEGs among mosquito species at each time point. At 1 dpi, 530 DEGs were common between *A. albopictus* and *A. malayensis*, representing 29% and 56% of all DEGs for each species, respectively. At 4 dpi, 130 DEGs were common, representing 7.3% and 13.8% of all DEGs for *A. albopictus* and *A. malayensis*, respectively. Overall, the transcriptomic impact of CHIKV infection is relatively conserved across the two mosquito species and a majority of DEGs at 4 dpi were already altered at 1 dpi.

We subsequently looked at the shared DEGs in *A. malayensis* infected with DENV or CHIKV at 1 and 4 dpi. Firstly, there was only one conserved DEG between all conditions (Fig. 1d) and it was the uncharacterized *AAFLPA_064235*, which was slightly upregulated in all conditions (Table S2). Secondly, we looked at the effect of time post infection. There were only two DEGs common between 1 and 4 dpi with DENV, which was less than between 1 and 4 dpi with CHIKV, as detailed above. Finally, we observed how the virus influenced the transcriptome within each time point. There were 28 DEGs common between DENV and CHIKV at 1 dpi, representing 38% and 3% of all DEGs for DENV and CHIKV, respectively. There were 19 shared DEGs between the two viruses at 4 dpi, representing 28% and 9% of DENV- and CHIKV-induced DEGs. Overall, in *A. malayensis*, DENV induced a different transcriptomic response at 1 and 4 dpi and the transcriptomic signature of DENV and CHIKV differed.

### Functional annotations of genes responsive to CHIKV and DENV infections in *A. albopictus* and *A. malayensis* midguts

**Figure 2:**
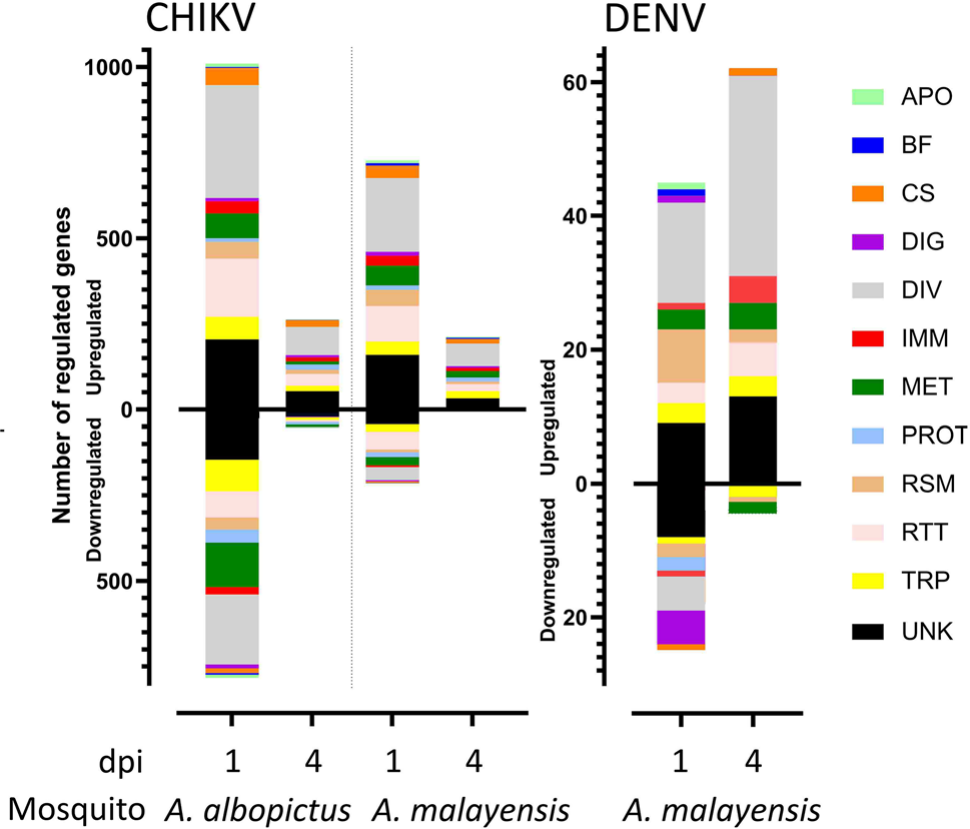
Distribution of the DEGs according to their functional group. APO, apoptosis; BF, bite and feeding, CS, cytoskeleton; DIV, diverse; DIG, digestion; IMM, immunity; MET, metabolism; PROT, proteolysis; RSM, redox, stress and mitochondria; RTT, replication, transcription and translation. TRP, transport; UNK, unknown.

We annotated the DEGs into different functional categories based on characterized functions, homology, or protein domains. For both comparative purposes and because of the absence of an annotated *A. malayensis* genome, we relied on the orthologs in *A. albopictus* to describe virus-induced gene alterations in *A. malayensis*. Apoptotic-related genes were mostly regulated by CHIKV in *A. albopictus* at 1 dpi (Fig. 2; Table S2) with the up- and downregulation of apoptosis-inducers such as CASP. Interestingly, the expression of an inhibitor of apoptosis (IAP; *AALFPA_076435*) was increased in all CHIKV conditions. Only one apoptotic gene was induced by DENV at 1 dpi in *A. malayensis*.

Several genes related to blood-feeding were regulated by CHIKV infection at the different time points and in both mosquito species but only one blood-feeding gene was regulated by DENV at 1 dpi in *A. malayensis* (Fig. 2; Table S2). Particularly, one putative odorant binding (*AALFPA_066142*) was strongly (> 48 fold) induced in all CHIKV conditions and one gustatory receptor (*AALFPA_047124*) was similarly highly upregulated by CHIKV at both time points in *A. malayensis*. Genes related to cytoskeleton were mostly upregulated in all the CHIKV conditions. However, contrasting regulation of cytoskeleton genes were observed for DENV in *A. malayensis*, where they were downregulated 1 dpi, but upregulated at 4 dpi (Fig. 2; Table S2). Genes related to digestion were mostly regulated by CHIKV at 1 dpi in both mosquito species and in lower quantity by CHIKV at 4 dpi and DENV at 1 dpi (Fig. 2; Table S2). None of the digestion-related genes were regulated by DENV at 4 dpi. Of interest, two collagen genes and one chitinase gene were upregulated in all CHIKV conditions. A large proportion of genes with diverse functions were regulated in all conditions.

Among the genes related to immunity, in CHIKV-infected *A. albopictus*, 36 DEGs were upregulated and 21 downregulated at 1 dpi, and 12 were upregulated at 4 dpi (Fig. 2; Table S2). In CHIKV-infected *A. malayensis*, 30 DEGs were induced and five were reduced at 1 dpi, while expression of nine were increased at 4 dpi. In DENV-infected *A. malayensis*, there was one upregulated immune DEGs and five downregulated at 1 dpi, and four increased DEGs at 4 dpi. Among these DEGs, there were several PRRs. One GNBP and three PGRPs were downregulated by CHIKV at 1 dpi in *A. albopictus*. A variety of serine protease or serine protease inhibitor DEGs were regulated in either direction by CHIKV and by DENV in both mosquito species. For IMD pathway, one activator (*sickie-like*; *AALFPA_052752*) was inhibited and another one (*hyperplastic discs*; *AALFPA_079063*) was activated at 1 dpi in CHIKV-infected *A. albopictus*. Another activator (*sickie-like*; *AALFPA_058168*) was upregulated by CHIKV at 4 dpi in both mosquito species. For Toll pathway, two Toll-like genes (*AALFPA_053631* and *AALFPA_057415*) were induced by CHIKV at 1 dpi in *A. albopictus*. For Jak/STAT pathway, three repressors (three Suppressor of cytokine signaling (SOCS)-like; *AALFPA_051177*; *AALFPA_054706*; *AALFPA_056404*) were activated at 1 dpi with CHIKV and at 4 dpi with DENV in *A. malayensis*, whereas one Bromodomain and WD repeat domain containing 3 gene (*BRWD3*, *AALFPA_050507*) was induced by CHIKV at 1 dpi in *A. albopictus*. For JNK pathway, two *Kayak-like* genes (*AALFPA_079058* and *AALFPA_079354*) were upregulated by CHIKV at 1 dpi in both mosquito species and by DENV at 4 dpi in *A. malayensis*. At 1 dpi with CHIKV, two other JNK activators were induced in either mosquito species (*A. albopictus*: *AALFPA_071724* and *AALFPA_078630*; *A. malayensis*: *AALFPA_071724* and *AALFPA_040889*). In terms of immune effectors, two complement genes (*AALFPA_045642* and *C1q*, *AALFPA_055182*) were increased by CHIKV at 1 dpi in *A. albopictus*, but no antimicrobial peptide (AMP) was directly regulated. Moreover, one helicase (*AALFPA_080443*), involved in RNAi, was induced at 1 dpi with CHIKV in *A. albopictus*. The same helicase plus another (*AALFPA_066096*) were upregulated at 1 dpi with CHIKV in *A. malayensis*. Overall, although we observed the regulations of components from several immune pathways, there was a consistent induction of JNK activators.

### Immune regulations in *A. albopictus* midgut infected by CHIKV

Because the immune response chiefly determines vector competence^32,33^, we focused on immunity. We identified the immune genes transcribed in *A. albopictus* midguts in both infected and control conditions, and classified them based on the categories defined by Palatini et al.^54^ (Table 1; Table S3). A majority of the immune genes identified in the *A. albopictus* genome were detected in our RNA-seq dataset (463 out of 664; not including JNK-related genes). Interestingly, most of the PRRs (GNBPs and PGRPs), all the components of the cytoplasmic signaling of the immune pathways (IMDPATH, TOLLPATH and JAKSTAT), 13 components of the JNK pathway, and three out of four REL downstream transcription factors were expressed in the midguts. Inversely, we detected a lower proportion of transcripts among enzymes involved in the regulation of the immune signaling, i.e., 63 of 118 CLIPs and 30 of 46 SRPNs. SPZ (nine of 13) and TOLL receptors (16 of 26) were also expressed in lower proportions. Effectors such as AMP, TEP and GALE had most of their orthologs expressed in midguts, whereas CTL, FREP, ML and PPO had lower proportions of orthologs detected. Members of ROS system were expressed in midguts with 1 out of 2 CATs, 33 out of 36 HPXs, 22 out of 28 SCRs and 5 out of 9 SODs. Moreover, a large proportion of SRRPs and most of APHAGs and apoptosis factors (CASPAs, CASPs and IAPs) were transcribed in midguts, completing the picture of the immune machinery activated in midguts.

**Table 1:** List of immune gene orthologs identified in *A. albopictus* and *A. malayensis* midgut.

Immune category	Abbreviations	*A. albopictus genome*^a^	Number expressed in midguts	
			*A. albopictus*	*A. malayensis*
Antimicrobial peptides	AMP	6	5	6
Autophagy pathway members	APHAG	30	29	14
Caspase activators	CASPA	3	3	3
Caspases	CASP	24	21	7
Catalases	CAT	2	1	3
CLIP-domain serine proteases	CLIP	118	63	9
C-type lectins	CTL	66	24	3
Fibrinogen-related proteins	FREP	52	35	6
Galectins	GALE	12	11	8
Gram-negative binding proteins	GNBP	13	12	2
Heme peroxidases	HPX	36	33	7
IMD pathway members	IMDPATH	16	16	9
Inhibitors of apoptosis	IAP	5	5	3
Jak/STAT pathway members	JAKSTAT	3	3	1
JNK pathway members	JNK	Not in Paper	13	11
Lysozymes	LYS	7	5	2
MD2-like proteins	ML	36	22	6
Peptidoglycan receptor-like proteins	PGRP	18	17	10
Prophenoloxidases	PPO	23	13	1
Rel-like NFkappa-B proteins	REL	4	3	2
Scavenger receptors	SCR	28	22	7
Serine protease inhibitors	SRPN	46	30	8
SiRNA pathway members	SRRP	51	45	20
Superoxide dismutases	SOD	9	5	2
Spaetzle-like proteins	SPZ	13	9	5
Thioester-containing proteins	TEP	7	5	1
Toll pathway members	TOLLPATH	10	10	4
Toll receptors	TOLL	26	16	2
Total		664	476	162

Gene identification was based on de novo assembled transcripts for *A. malayensis.*

^a^Data extracted from^54^.

To identify patterns in the immune response to CHIKV infection at 1 and 4 dpi in *A. albopictus*, we clustered and plotted the expressions of the 50 most variable immune genes in a heatmap (Fig. 2; Table S3). We detected 5 clusters. Cluster 1 contained only one C-type lysozyme that also belonged to the significant DEGs identified above and was highly upregulated (> 77 fold) at both time points. Cluster 2 was made of five genes only downregulated at 4 dpi and included two PRRs (one PGRP and one GNBP), two proteases involved in the regulation of the immune signaling (one CLIP and one SRPN), and one LYS. Cluster 3 consisted of 12 genes induced at 1 dpi and then reduced at 4 dpi. These included several genes implicated in regulating the immune activation, i.e., three SRPNs (two of which were significant DEGs), one SPZ and one TOLL. Their clustering with effectors such as one AMP (Attacin-B), one CTL and two FREPs, suggested their regulations through the above enzymes. Cluster 3 also included two SCRs involved in ROS regulation and one PPO that induces melanization. Inversely to cluster 3, cluster 4 was made of 14 genes downregulated at 1 dpi and upregulated at 4 dpi. Among the proteins that regulate immune signaling, there were one PGRP, one SPZ, one SRPN and one CLIP. Six immune effectors were also grouped in the same cluster and included three FREPs and three MLs. Three CASPs that trigger apoptosis were present in cluster 4, together with one SRRP. Finally, cluster 5 contained 18 genes only downregulated at 1 dpi. Genes involved in immune signaling regulation were two PGRPs, one TOLL, three SRPNs and two CLIPs. One ML effector was also present. Regulations of ROS was apparent due to the clustering of five HPXs, regulation of melanization due to two PPOs, and regulation of RNAi because of two SRRP aubergine homologs.

### Immune regulations in *A. malayensis* midgut infected by CHIKV and DENV

In *A. malayensis*, we undertook a similar immune-centered approach. However, to provide the most accurate description for this mosquito species that does not have an assembled genome, we de novo assembled transcripts before searching for protein homologs in *A. albopictus* transcriptome. We identified 162 immune proteins (Data S1 details the protein sequences) expressed in the midgut of *A. malayensis* and functionally categorized them as above^54^ (Table 1; Table S4). There were a lower number of expressed homologs for all immune categories than in *A. albopictus*, except for AMP. These lower numbers of transcripts may be due to the conservative parameters (see “Methods”) we used for homolog identification. Nonetheless, for immune signaling, we found 12 PRRs (2 GNBPs and 10 PGRPs), 5 SPZs, 2 TOLLs, 9 components of the IMDPATH, 1 of the JAKSTAT, 11 of the JNK, 4 of the TOLLPATH and 2 REL homologs. Among enzymes that regulate the signaling, we detected 9 CLIPs and 8 SRPNs. For effectors, we noted 6 AMPs, 3 CTLs, 6 FREPs, 8 GALEs, 2 LYSs, 6 MLs and 1 TEP. Moreover, our results indicated a functional RNAi with 20 SRRPs. Autophagy, apoptosis, melanization and ROS regulation were also activated with 14 APHAGs, 13 apoptosis-related (3 CASPAs, 7 CASPs, 3 IAPs), 1 PPO, and 19 oxidative stress-related transcripts (3 CATs, 7 HPXs, 7 SCRs and 2 SODs), respectively.

We next clustered the expression of the 50 most variable immune genes upon CHIKV (Fig. 4a; Table S5) and DENV (Fig. 4b; Table S5) infection, separately. With CHIKV-infected samples, we identified four clusters. Cluster 1 contained ten genes only upregulated at 1 dpi. Among the components of the immune pathway signaling, there were two SRPNs, one JNK component, and one REL transcription factor. For effectors, one LYS, one FREP and one GALE were clustered. The detection of two CASPs and Ago2, a major SRRP, suggested apoptosis and RNAi activation, respectively. Cluster 2 was made of 16 genes induced at 1 dpi and then reduced at 4 dpi. For signaling regulation, there were one GNBP, three SPZs, one CLIP and one homolog of Kayak, the JNK transcription factor. Multiple effectors were in cluster 2 and included two AMPs, one TEP, one FREP, one LYS, one GALE and one ML. There were also one APHAG and two SCRs. Cluster 3 was composed of eight genes upregulated only at 4 dpi. For immune signaling, there were one GNBP, one TOLL and one CLIP. For effectors, there was only one FREP. Additionally, there were two ROS-related genes (one SOD and one SCR), one IAP and one APHAG. Cluster 4 consisted of 16 genes only downregulated at 1 dpi. Those related to immune signaling included one CTL, two SRPNs, one IMDPATH and Cactus, the negative regulator of TOLLPATH transcription. Among effectors, there were two MLs. One APHAG and three HPXs were also clustered. Interestingly, five SRRPs were grouped in cluster 4 and included Ago3, loquacious and two aubergine homologs.

With DENV-infected samples, we identified five clusters (Fig. 4b; Table S5). Cluster 1 consisted of six genes moderately upregulated at 1 dpi and strongly upregulated at 4 dpi. Among signaling genes, there was only one JNK component. Effectors included one ML and one GALE. There were also one CASPA, one APHAG and one SCR. Cluster 2 included 11 genes only upregulated at 4 dpi. No immune signaling-related genes were in cluster 2 and there were one ML and one GALE effectors. There were three apoptosis-related genes (two IAPs and one CASP), two APHAGs, one SOD and three RNAi-related genes (SRRPs). Cluster 3 was made of three genes induced at 1 dpi and then reduced at 4 dpi. These included one AMP, one SPZ and one LYS. Cluster 4 consisted of seven genes only upregulated at 1 dpi. Immune signaling genes included one GNBP and one SPZ, while immune effectors were one TEP, three FREPs and one AMP. Eventually, cluster 5 was composed of 23 genes moderately upregulated at 1 dpi and moderately downregulated at 4 dpi. Immune signaling genes included two PGRPs, three SPZs, three CLIPs, two SRPNs and two IMDPATHs such as an *IKK*-*β* homolog. Effectors consisted of two AMPs, three MLs and one CTL. There were also one APHAG, two CASPs and two ROS-related genes (one CAT and one SCR).

## Discussion

Public health threats from dengue and chikungunya are expanding geographically and in intensity^3,56^. While *A. aegypti* remains the main vector, other *Aedes* species are competent enough to trigger moderate-scale epidemics and sustain DENV and CHIKV transmissions^8,57^. Furthermore, the ongoing deployment of new interventions aimed at *A. aegypti* like the *Wolbachia*-mediated transmission reduction^58,59^ will most probably promote the roles of these secondary vectors. Here, we set to prepare for the next steps of vector control by comprehensively describing the transcriptomic responses to CHIKV and DENV in two capable vectors, *A. albopictus* and *A. malayensis*^27^. Because infection onset in the mosquito midgut dictates vector competence, we analyzed midguts at early times post infection, i.e., 1 and 4 dpi. Our results profile DEGs in two mosquito species infected by two viruses at two time points. Next, we focused on immune-related genes and utilized a de novo transcriptome assembly in *A. malayensis* to identify immune genes for the first time in this species. Overall, our study reveals patterns of transcriptomic responses in two *Aedes* vectors and details the immune responses, shedding new light on shared and divergent mechanisms of arboviral transmission.

The overall transcriptomic response was shaped by viral infections as well as time post infection and varied between the two mosquito species. Although we orally infected mosquitoes with a similar inoculum for both viruses, the transcriptomic response was greater upon CHIKV than DENV infection. Such difference may relate to the faster CHIKV replication kinetic^52^, which reached a plateau as early as 1 dpi in both mosquito species. However, in spite of the similar viral genomic RNA (gRNA) levels between the two time points, the number of CHIKV-induced DEGs did not correlate with gRNA copies across the time points and sharply decreased from 1 to 4 dpi. This suggests a return to homeostasis after quick establishment of viral replication factories. Interestingly, CHIKV-induced DEGs at 4 dpi were mostly already regulated at 1 dpi in both mosquito species. These conserved regulated genes may represent the cellular machinery required for CHIKV multiplication. In DENV-infected *A. malayensis* midguts, the transcriptomic response did not correlate with gRNA quantities, which grew significantly between 1 and 4 dpi whereas DEG numbers were similar between the time points. In contrary to CHIKV infection, there was a very limited number of shared DEGs between 1 and 4 dpi in DENV-infected *A. malayensis*. There was also little overlap between DENV- and CHIKV-induced DEGs in this mosquito species. These transcriptomic differences document divergences in how DENV and CHIKV establish infection in mosquito midguts.

**Figure 3:**
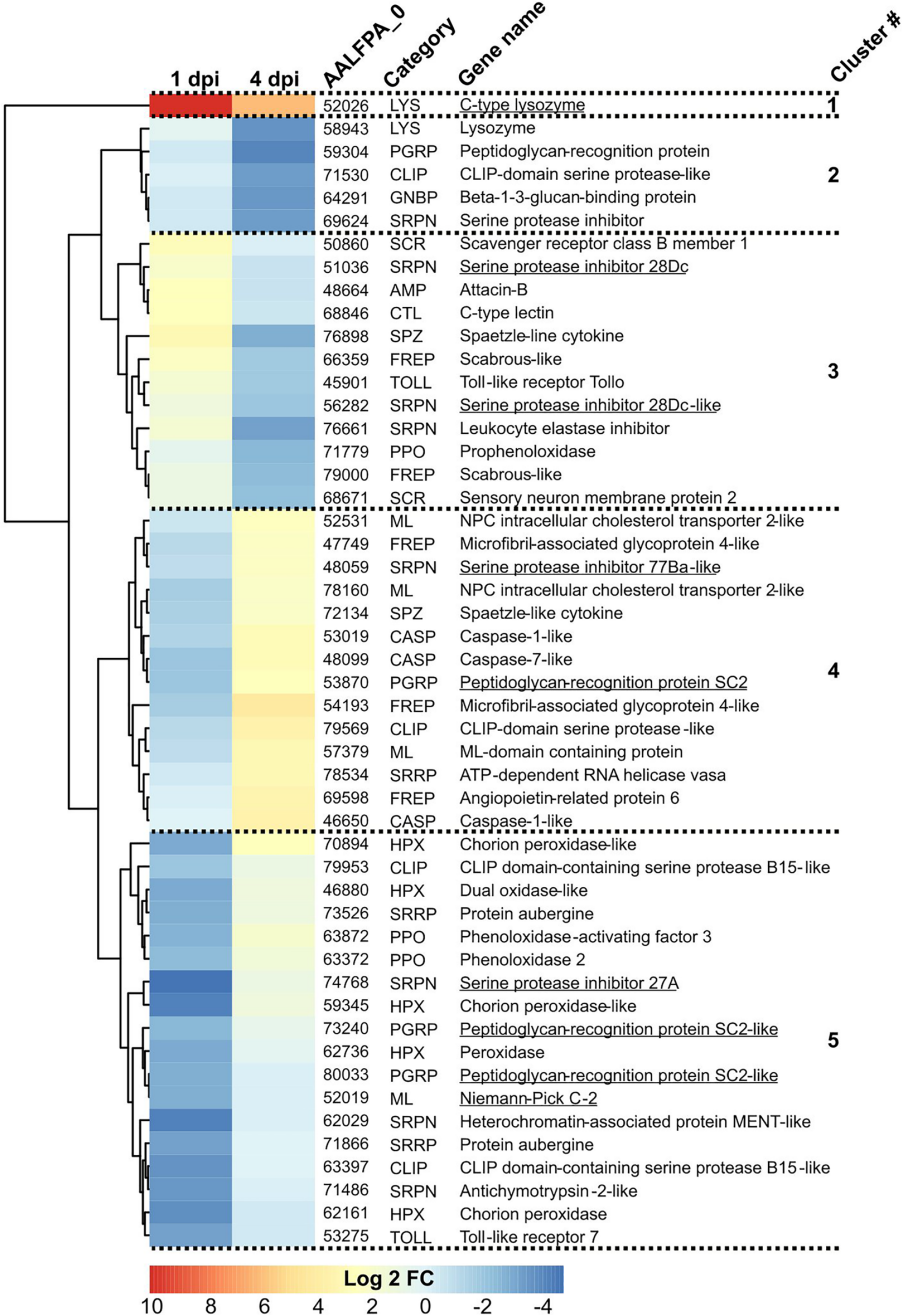
Immune gene regulation upon CHIKV infection in *A. albopictus* midgut at 1 and 4 dpi. Heatmap of the top 50 most regulated DEGs. Underlined genes were significantly regulated. Hierarchical clustering of gene expression is shown on the left.

**Figure 4:**
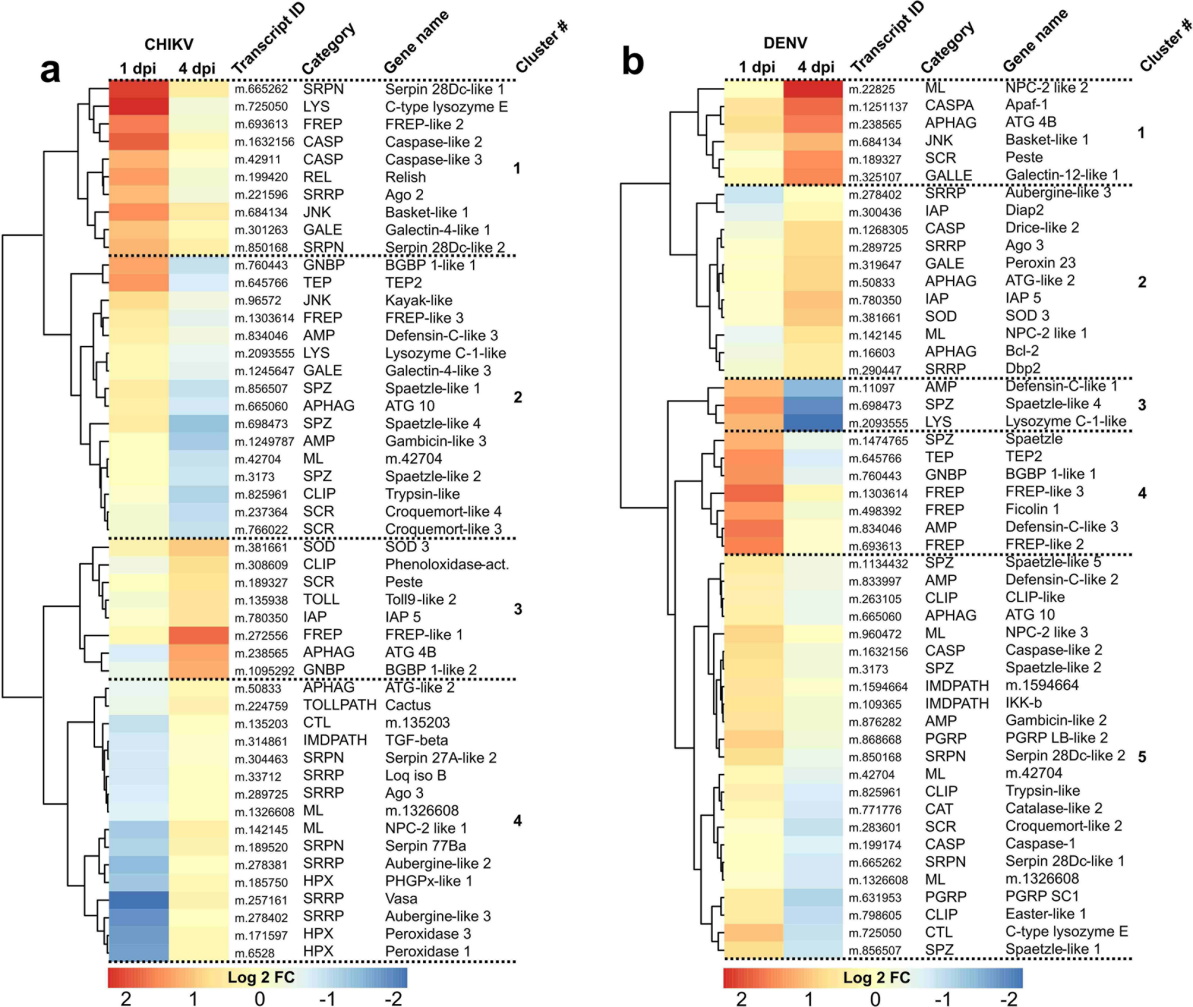
Immune gene regulation upon CHIKV and DENV infection in *A. malayensis* midgut at 1 and 4 dpi. Heatmap of the top 50 most regulated DEGs. None of these gene were significantly regulated. Hierarchical clustering of gene expression is shown on the left.

Strikingly, we identified a transcriptomic signature of CHIKV infection. There were 81 DEGs regulated in the same direction upon CHIKV infection in the two mosquito species (comparison was made using homologs from both species) and at both time points (Fig. 1; Table S2). While these common DEGs were related to multiple functional groups, we discuss the top eight most regulated ones as they were highly induced in all conditions. AALFPA_052026 and its *A. malayensis* homolog have a *D. melanogaster* homolog (CG8492) that reduces viral infection^60^. When we detailed the immune response, this gene did not cluster with other immune genes in *A. albopictus* (Fig. 3). However, in *A. malayensis*, it responded similarly to a Relish homolog (Fig. 4a), suggesting a potential regulation through one of the immune pathways that induces Relish. A deubiquitinase was part of the CHIKV transcriptomic signature and may have influenced the immune response and cellular homeostasis, given the function of ubiquitination in both processes^61,62^. CHIKV infection consistently induced a serine-rich protein kinase (SRPK), which may influence gene expression through chromatin remodeling^63^. A cytochrome P450 monooxygenase was upregulated and could have metabolized xenobiotic substances^64,65^. Interestingly, two highly expressed genes of the CHIKV signature are related to cellular development, i.e., a homolog of hunchback, a transcription factor that regulates cellular development^66,67^, and a homolog of canoe, which maintains adherens junction between cells. Both genes may be required for the cell multiplication that occurs in infected mosquito midguts to compensate for damaged cells^68^. Together with two other uncharacterized homologs, these top induced DEGs shared across all our CHIKV conditions deserve further functional studies.

Given the importance of midgut immunity in determining vector competence^30,32,33^, we detailed the immune response in both mosquito species. In *A. albopictus*, most of the components of the IMD, TOLL, JAK/STAT and JNK pathways were expressed, indicating that the corresponding pathways are functional in midguts. In contrary, a lower proportion of enzymes involved in signaling regulation were detected and this suggests a midgut-specific regulation of immunity as different CLIP or SRPN interact with different immune components^69,70^. Apoptosis, autophagy, RNAi, ROS and melanization were also functional in midguts. In *A. malayensis*, smaller proportions of the immune pathway components were detected in midguts. While this may be related to the technical limitations associated with de novo assembly such as high error rates and short assembled contigs^71^, each canonical pathway was still represented by several key members. For TOLL and IMD pathways, the two downstream transcription factors, homologs of *D. melanogaster* Dorsal and Relish, respectively, were expressed in *A. malayensis* midguts (Table S4). The negative regulators for *Dorsal* and *Relish*, namely Caspar and Cactus respectively, were also found. Similarly, the downstream transcription activators for JNK and JAK/STAT, i.e., *Kayak* and *STAT* respectively, were transcribed, confirming the conservation of these pathways across insects^72^ and indicating that the canonical immune pathways function in *A. malayensis* midguts.

Activation of the TOLL, IMD, JAK/STAT and JNK pathways occurs through transcriptional regulation of their components in response to infection^34,43,73,74^. Interestingly, the JNK pathway appeared to be induced in both mosquito species and upon both viral infections. Two *Kayak-like* homologs were significantly upregulated (i.e., DEG) at 1 dpi with CHIKV in both mosquito species and at 4 dpi with DENV in *A. malayensis*. In *A. aegypti* salivary glands, *Kayak* was previously shown to be induced by both CHIKV and DENV and to be co-regulated with *Basket*, one of the upstream components of the JNK pathway^34^. Supporting the activation of JNK, we also observed an induction of the *Basket* homolog (among the 50 most regulated immune genes) in *A. malayensis* at 1 dpi with CHIKV and 4 dpi with DENV. JNK activation clears viral infection by activating apoptosis and the complement system^34^. Together with the JNK components, we observed co-regulations of multiple CASPs and TEPs. Although our transcriptomic data indicate an activation of the JNK pathway, other immune pathways appeared induced as well. For instance, the induction of a *Relish* homolog together with the reduction of its inhibitor *Cactus* among the most regulated immune genes in *A. malayensis* at 1 dpi with CHIKV suggest the activation of the Toll pathway. The induction of *Ikk*-*β* in DENV-infected *A. malayensis* at 1 dpi show activation of the IMD pathway^75^. However, while components of several immune pathways were regulated, the activation of the JNK pathway was consistent in both *A. albopictus* and *A. malayensis* upon infection by CHIKV and DENV.

In conclusion, we conducted a high-throughput transcriptomic analysis in two *Aedes* vectors with DENV and CHIKV at two early time points in midguts. Our dataset provides a multidimensional picture of the transcriptomic and more specifically immune regulations in these conditions. In *A. albopictus*, we identified different gene regulation clusters, which may represent different immune pathway responses to CHIKV infection in midguts. In *A. malayensis*, we provided the first description of the immune response to arboviral infections. This knowledge is required to broaden our understanding of arboviral transmission by *Aedes* vectors.

## Material and methods

### Mosquito rearing

*Aedes albopictus and A. malayensis* mosquito colonies were established in 2010 and 2014, respectively, from eggs collected using oviposition traps in the parks of Singapore^27^. Since then, these colonies were reared in the insectary where eggs were hatched in MilliQ water, larvae fed on a mixture of fish food (TetraMin fish flakes), yeast and liver powder (MP Biomedicals) and adults maintained on 10% sucrose and fed pig’s blood twice weekly. Mosquito colonies were maintained at 28 °C and 50% relative humidity with a 12 h:12 h light: dark cycle.

### Viruses

Dengue virus serotype 2 PVP110 was isolated from a patient enrolled in the Early DENgue infection and outcome study (EDEN) conducted in Singapore in 2008^76^. Chikungunya virus SGP011 was isolated from a patient at the National University Hospital in Singapore in 2008^50^. DENV isolates were propagated in C6/36 (CRL-1660) and CHIKV in Vero (CCL-81) cell lines. Virus stocks were titered with BHK-21 cell plaque assay as previously described^77^, aliquoted and stored at − 80 °C. Virus stocks were thawed only once for infection.

### Mosquito infection

Three-to-five day-old female mosquitoes were starved for 24 h before they were fed on an infectious blood meal containing 40% volume of washed erythrocytes from specific pathogen free (SPF) pig’s blood (PWG Genetics), 5% 10 mM ATP (Thermo Scientific), 5% human serum (Sigma) and 50% virus solution in RPMI media (Gibco), using Hemotek membrane feeder system (Discovery Workshops) covered with porcine intestine membrane (sausage casing). The virus titers in blood meals were 2 × 10^7^ pfu/ml for DENV and 1 × 10^7^ pfu/ml for CHIKV. Blood titers were validated by plaque assay using BHK-21 cells. Control mosquitoes were fed with the same blood meal composition except for virus solution, which was replaced by RPMI. Following oral feeding, the fully engorged females were selected and kept in a cage with ad libitum access to a 10% sucrose solution in an incubation chamber with conditions similar to insect rearing.

### Detection of Wolbachia

Groups of 10 mosquitoes were homogenized using a bead mill homogenizer (Biospec Products) and total DNA was extracted with QIAamp DNA extraction kit (Qiagen). *Wolbachia* presence was detected by PCR amplification with GoTaq Master Mix (Promega) and the following primers: Forw-CAT ACC TAT TCG AAG GGA TAG; Rev-AGC TTC GAG TGA AAC CAA TTC^78^. Two replicates were conducted for *A. albopictus* and *A. malayensis* colonies.

### Midgut collection, library preparation and RNA-sequencing

Midguts from control, DENV- and CHIKV-orally infected *A. albopictus* and *A. malayensis* were dissected at one- and four-days post infection (dpi). The blood was removed from the midguts. Three repeats per condition with 20 midguts per replicate were homogenized using a bead mil homogenizer (Biospec Products, USA). Total RNA was extracted using E.Z.N.A Total RNA kit I (OMEGA Bio-Tek). RNA-sequencing libraries were prepared using a True-Seq Stranded Total RNA with Ribo-Zero Gold kit (Illumina), according to manufacturer’s instructions. Following quantification by RT-qPCR with a KAPA Library Quantification Kit (KAPA Biosystems), libraries were pooled in equimolar concentrations for cluster generation on cBOT system (Illumina) and sequenced (150 bp pair-end) on a HiSeq 3000 instrument (Illumina) at the Duke-NUS Genome Biology Facility.

### Identification of differentially expressed genes

Sequenced read quality was assessed using the Java program FastQC (Babraham Institute Bioinformatics, UK). Contaminating adaptors and low-quality reads (Phred + 33 score < 20) were removed with Trimmomatic v0.39^79^ using a sliding window of 4 bps. To confirm virus infection and mosquito species, reads were aligned with Bowtie2 v2.3.4.3^80^ to the genomes of the viruses used for infection and *A. albopictus* and *A. malayensis* mitochondrial cytochrome c oxidase 1 (CO1) genes, respectively. Reads from both mosquito species were aligned on *A. albopictus* genome (AaloF1.2) and the *A. aegypti* genome (AaegL5.0) with STAR v2.5.4a in two- pass mode^81^ and HTseq v0.6.0^82^ used to produce count files, that were then used as input into DESeq2^83^ to identify DEGs with at least a 1.4-fold (log 2 = 0.48) change between control and infected conditions and an adjusted False Discovery Rate (FDR) of 0.05. DEGs were annotated by importing gene names, descriptions and computed GO functions and processes from VectorBase^84^, and functional groups were manually assigned. Predicted protein sequences were also searched against the NCBI Diptera protein database (Blastp, e-value 1.0E−5 threshold) and the FlyBase^85^ *D. melanogaster* peptide database for identification of orthologues.

### Validation of RNA-seq by real-time quantitative PCR

*Aedes albopictus* and *A. malayensis* mosquitoes from different batches than the ones used for RNA-seq were orally infected with DENV and CHIKV. At four dpi, midguts from eight mosquitoes were dissected and pooled in triplicates. Total RNA was extracted from the midgut samples using a E.Z.N.A. Total RNA kit I (OMEGA), DNAse treated using a Turbo DNA-free kit (Thermo Fisher Scientific), and reverse transcribed using iScript cDNA synthesis kit (Biorad). Expression for 11 genes was quantified with qPCR using the SensiFast Sybr no-rox kit (Bioline) and primers designed based on *A. albopictus* genome, as detailed in Table S6. *Actin* expression was used for normalization. Reactions were performed with the following cycle conditions: an initial 95 °C for 10 min, followed by 40 cycles of 95 °C for 5 s, 60 °C for 20 s and ending with a melting curve analysis. The delta delta method was used to calculate relative fold changes. Pearson correlations were calculated between qPCR-based and DEG log2 fold changes with excel.

### De novo identification of immune genes in *A. malayensis* and quantification of their expression

For the de novo assembled *A. malayensis* transcriptome, reads were assembled with Trinity v2.4.0^86^ and ABySS v2.1.5^87^. Trinity parameters specified a contig size of at least 150 bps with a minimum k-mer coverage of 2. ABySS assemblies were completed for k-mer sizes 21, 31, 41, 51, 61, 71, 81 and 91 bps. Individual assemblies were then combined and redundancy removed with CD-HIT v4.6.8^88,89^. Blastn v2.12^90^ was used to identify contigs belonging to the DENV2 and CHIKV genomes used for infection, and these were subsequently removed. Transdecoder from the Trinity pipeline was then used to identify all contigs with open reading frames (ORFs), and only those with complete and nonredundant ORFs were retained. Blastp was then used to identify translated contigs matching (e-value 1.0E^−5^) to NCBI Diptera proteins (taxid:7147, accessed June 2021), and all others were filtered out. Using RSEM v1.3.0^91^, reads were mapped to this final set and used as input into DESeq2^83^ to determine log2 Fold-changes between infected and control conditions and DEGs with the same criteria as detailed above.

To confidently annotate all *A. malayensis* immune transcripts, the protein products of *A. albopictus* immune genes identified by Palatini et al*.*^54^ and an additional 13 JNK pathway genes were used as a database to identify orthologues from the translated *A. malayensis *de novo assembled transcriptome. This was done with Diamond Blastp^92^ and a strict search criteria of an e-value 1.0E^−10^ or less, followed by a second search against all annotated *A. albopictus* proteins to confirm identifications. From this identified set, only transcripts that could be fully translated with no ambiguities, covered at least 90% of database subject sequences, and shared less than 98% identity with each other were considered confident *A. malayensis* immune genes. These were also searched against the FlyBase^85^ *D. melanogaster* peptide database for identification of orthologues.

## Supplementary Information

Supplementary Information 1.

Supplementary Table S1.

Supplementary Table S2.

Supplementary Table S3.

Supplementary Table S4.

Supplementary Table S5.

Supplementary Information 7.

## Data Availability

Sequenced reads generated during this study are available under NCBI accessions: SRR14621613-SRR14621644, BioProject PRJNA731987.
