# Supplementary material for: Midgut transcriptomic responses to dengue and chikungunya viruses in the vectors Aedes albopictus and Aedes malayensis
Source: Sci Rep. 2023 Jul 12;13:11271. doi: 10.1038/s41598-023-38354-9 (PMC10338677; doi:10.1038/s41598-023-38354-9)
Supplement: Supplementary file 7 — Supplementary Information 7. [file 41598_2023_38354_MOESM7_ESM.docx]

**Supplementary Information**


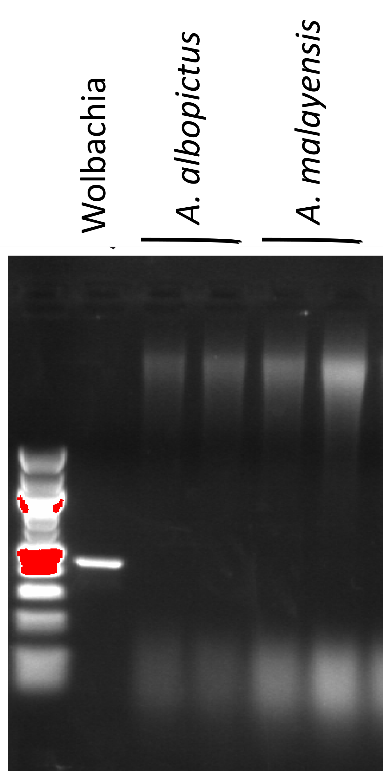


**Figure S1. Detection of Wolbachia in *A. albopictus* and *A. malayensis* colonies.**


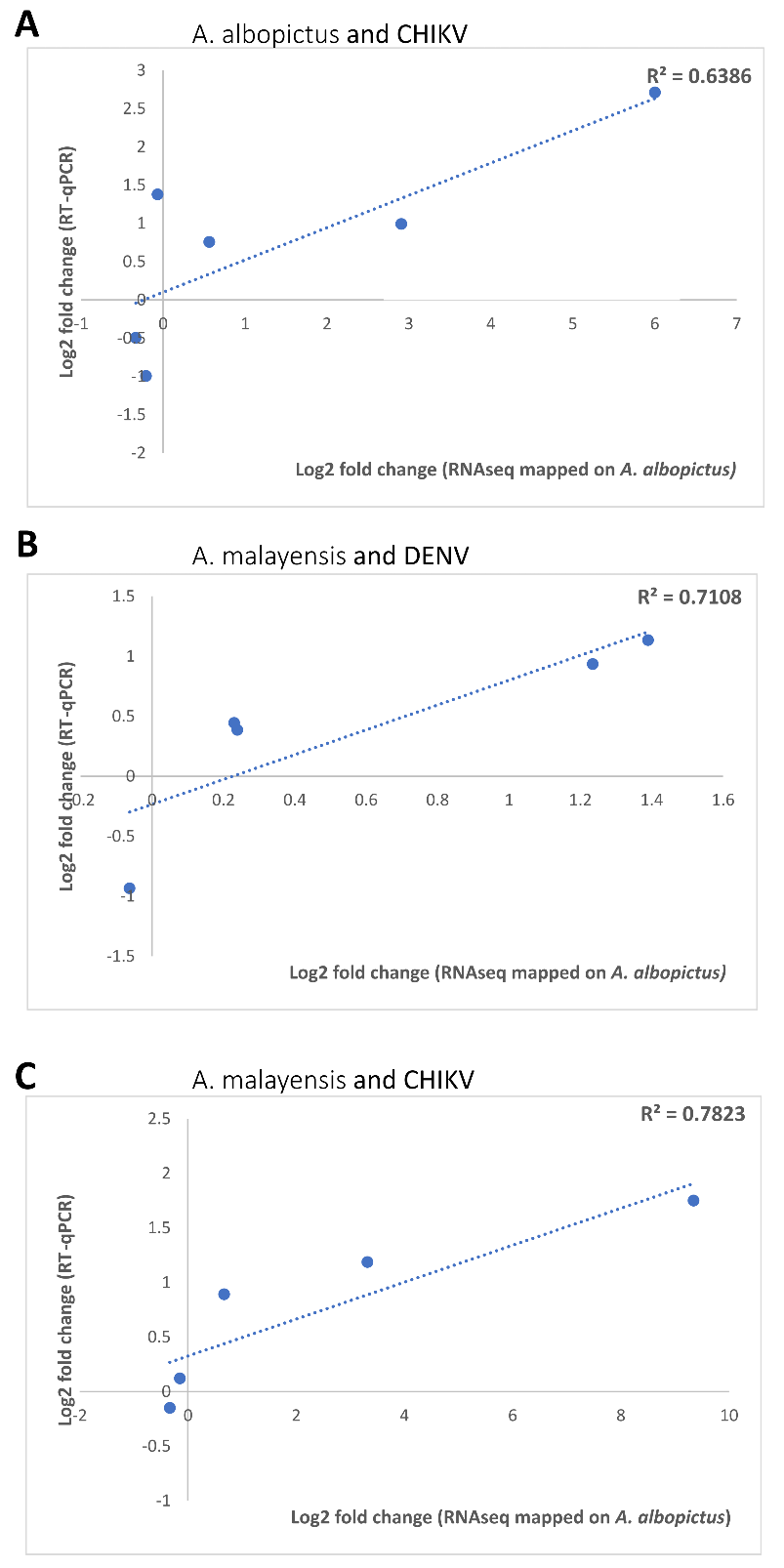


**Figure S2. Validation of RNAseq data mapped on *A. albopictus* by RT-qPCR.**

Gene expression comparisons for 6 genes as determined by RNA-seq mapping to the *A. albopictus* genome and by RT-qPCR. Pearson correlation is indicated for each comparison.

**
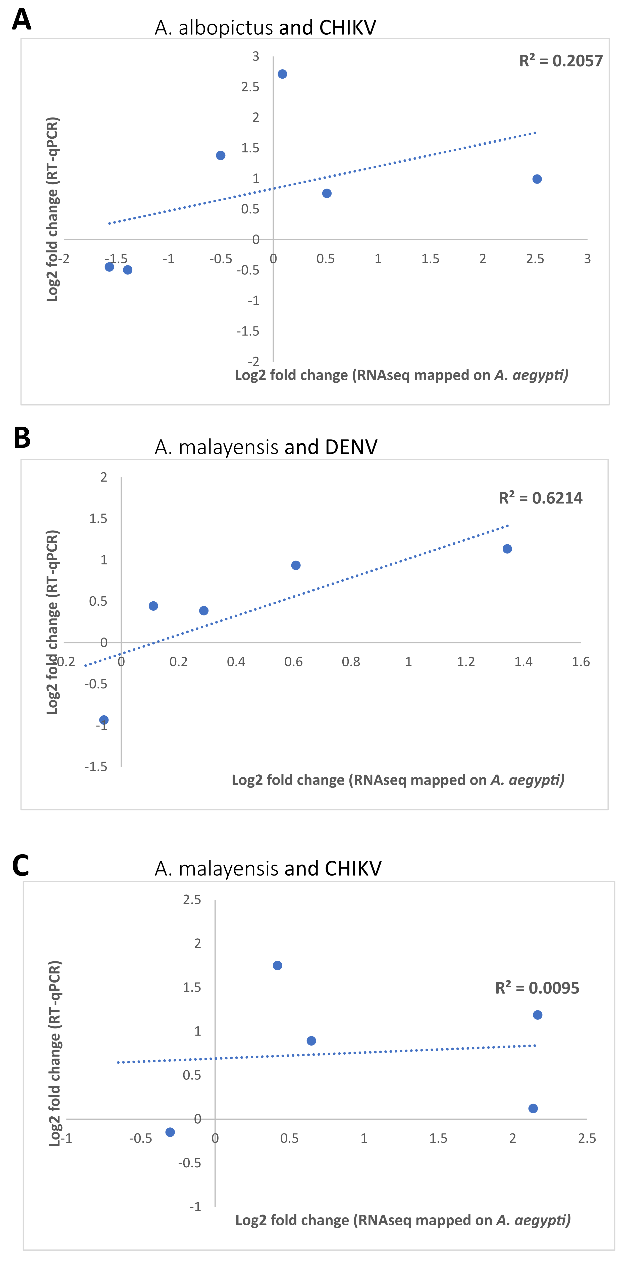
**

**Figure S3. Validation of RNAseq data mapped on *A. aegypti* by RT-qPCR.**

Gene expression comparisons for 6 genes as determined by RNA-seq mapping to the *A. aegypti* genome and by RT-qPCR. Pearson correlation is indicated for each comparison.

**Table S1. Percentages and number of reads homologous to CHIKV and DENV, and to cytochrome c oxidase (COI) from *A. albopictus* and *A. malayensis***

**Table S2. DEGs for all conditions based on mapping onto *A. albopictus* genome**

Log2 fold change (Log2FC) is provided for each DEG. VectorBase description, GO Function, GO Process, Gene type, UniProt Function and description of *D. melanogaster* homologs are provided to determine the gene function.

**Table S3. Regulation of all immune genes upon CHIKV infection in midguts of *A. albopictus* at 1 and 4 dpi**

Immune categories as in^54^

**Table S4. Identification of immune genes in *A. malayensis* midguts through *de novo* assembly**

Identification was based on *de novo* transcripts.

**Table S5. Regulations of all immune genes upon CHIKV and DENV infection in midguts of *A. malayensis* at 1 and 4 dpi.**

Transcripts were identified from *de novo* assembly. ^1^Gene name is based on identification as in Table S4.

**Table S6. Primers used for RT-qPCR.**

| Gene ID | Forward | Reverse |
| --- | --- | --- |
| AALFPA_068948 | CCATCAGCACATGTGGCATC | CAGGATTGCGTGGAGCTTAC |
| AALFPA_050898 | CTAGTATCGGAACGCTGGGT | AACGGTGACCGGATTGTACT |
| AALFPA_075385 | CGTGACGAGGAGGAACCGAA | TCTGCCCGAAGCTAGTCAGC |
| AALFPA_048972 | GACGGTCTGGTCAGCATAGA | ACACTGACGAGCTGGTTGTA |
| AALFPA_049960 | GCCAGAGTGTTCTCGGTCAA | CGTATCGTGGTGAAACCGGA |
| AALFPA_047102 | TGTTACACCATGACCCGTGG | GTACTCCTGGTACGGGGTCT |
| AALFPA_068228 (Actin) | GAACACCCAGTCCTGCTGACA | TGCGTCATCTTCTCACGGTTAG |

**Data S1. Protein sequences for immune genes in A. malayensis identified by de novo assembly**
